# The irreplaceable nature of care: the art of caring in times of technology and connectivity

**DOI:** 10.47626/1679-4435-2026-1463

**Published:** 2026-02-12

**Authors:** Patrícia Simonaggio

**Affiliations:** 1 Occupational Physician, independent practitioner (no institutional affiliation), Porto Alegre, RS, Brazil

**Keywords:** occupational medicine, absenteeism, empathy, technology, comprehensive health care., saúde ocupacional, absenteísmo, empatia, tecnologia, assistência integral à, saúde.

## Abstract

This article reflects on the role of human care in occupational health management within a context shaped by hyperconnectivity and the growing integration of technologies. In organizational settings driven by efficiency and productivity, care is reaffirmed as a structuring axis for more humane, sustainable, and effective labor relations. Despite advances in digital tools and artificial intelligence, care remains irreplaceable. Its relevance to preventing absenteeism, promoting mental health, and strengthening worker engagement is highlighted. Accordingly, the article advocates an articulation between technology and humanization, in which care emerges as a competitive differentiator and a cornerstone of more empathetic and effective management.

## Abstract (pt)

Este artigo propõe uma reflexão sobre o papel do cuidado humano na gestão da saúde ocupacional em um contexto marcado pela hiperconectividade e pela crescente incorporação de tecnologias. Em um cenário organizacional orientado pela eficiência e pela produtividade, resgata-se o cuidado como eixo estruturante de relações de trabalho mais humanas, sustentáveis e eficazes. Argumenta-se que, apesar dos avanços das ferramentas digitais e da inteligência artificial, o cuidado permanece insubstituível. Destaca-se sua relevância para a prevenção do absenteísmo, a promoção da saúde mental e o fortalecimento do engajamento dos trabalhadores. Defende-se, assim, uma articulação entre tecnologia e humanização, na qual o cuidado se configura como diferencial competitivo e pilar de uma gestão mais empática e efetiva.

## INTRODUCTION

In the contemporary landscape of occupational health, technological advances, particularly artificial intelligence (AI), have increasingly transformed professional practice, ranging from diagnostic processes to the management of workers’ health. Despite these significant advances, human care remains irreplaceable. The concept of care extends beyond technical and procedural dimensions and encompasses the construction of bonds, active listening, and an understanding of workers’ individual needs. This article examines the role of AI in occupational health and emphasizes the importance of preserving a humanized dimension of care as an essential component of therapeutic and management strategies.

### THE IMPACT OF ARTIFICIAL INTELLIGENCE ON OCCUPATIONAL HEALTH

AI has been widely employed to optimize diagnostic accuracy, predict health risks, and support occupational health management. AI-based systems have demonstrated effectiveness in the early identification of conditions such as chronic diseases and mental disorders, often before overt clinical manifestations occur. Algorithms capable of analyzing large volumes of workers’ health data enable the identification of patterns associated with absenteeism risk or the need for early interventions [^1^].

However, while AI contributes to gains in efficiency and diagnostic precision, it does not replace the empathic dimension of human care. Care is not limited to data analysis; it requires an understanding of emotions, life experiences, and the sociolabor context of workers. Active listening, welcoming attitudes, and empathic presence remain essential elements for promoting mental and physical health in the workplace.

### HUMANIZATION IN THE CONTEXT OF OCCUPATIONAL MEDICINE

Human care transcends the technical role of health professionals. In occupational medicine in particular, care must be understood in a comprehensive manner, encompassing workers’ physical, emotional, social, and psychological well-being. The relationship between the health professional and the worker should not be merely transactional but grounded in respect, trust, and mutual understanding.

The humanization of care in occupational health is fundamental to improving treatment adherence and reducing stigma associated with mental disorders and other health conditions. Professionals who demonstrate empathy and sensitivity contribute to the creation of a climate of trust, in which workers feel safe to express concerns and seek support when needed [^2^]. Evidence further indicates that humanized care practices are associated with better health outcomes and greater satisfaction in the work environment [^3^].

### CARE AS A PILLAR OF ORGANIZATIONAL SUSTAINABILITY

Care in the workplace extends beyond clinical assistance and assumes a strategic role in organizational health management. In the face of growing challenges related to mental health, burnout, and increasing absenteeism, valuing care as a daily and systemic practice becomes essential. Organizations that recognize workers as individuals rather than merely as resources tend to achieve better outcomes in engagement, productivity, and talent retention.

Beyond its ethical value, care constitutes a competitive advantage. Its implementation requires active listening, continuous organizational assessment, the development of empathic leadership, and a firm commitment to promoting comprehensive health. From this perspective, humanized care models should be incorporated into management policies as a structural element of sustainability and corporate responsibility.

This understanding represents the core of an occupational health management approach that goes beyond traditional models. Effective transformation occurs when organizational leadership recognizes that care is not a cost, but one of the primary assets for long-term success. Promoting care involves ensuring a safe work environment, valuing mental health, and strengthening workers’ sense of belonging. Absenteeism management, for example, goes beyond merely counting absences and requires an understanding of its determinants, such as demotivation, emotional overload, or inadequate working conditions.

### ARTIFICIAL INTELLIGENCE AS AN ALLY OF CARE

Although AI represents a powerful tool, it should be understood as a complement to human care rather than a substitute. AI can enhance data management, improve the accuracy of health risk identification, and support the personalization of occupational health strategies according to workers’ individual needs. However, it is not capable of capturing the emotional and psychological nuances inherent to the human experience, which are central to occupational health.

AI may, for example, assist in monitoring workers’ mental health by identifying behavioral patterns associated with stress or burnout. Nevertheless, human intervention remains indispensable for interpreting these data and providing appropriate emotional support. The integration of AI with human care maximizes the benefits in occupational health by ensuring that decisions are guided not only by data, but also by understanding and empathy [^4^].

The World Health Organization emphasizes the importance of applying ethical principles and human rights standards to the use of AI in health, highlighting that such technologies must be developed and implemented responsibly, with a focus on safety and individual well-being [^5^]. Similarly, the International Labour Organization recognizes that AI and digital technologies are transforming occupational safety and health management, requiring continuous adaptation by professionals in the field [^6^].

The United States National Institute for Occupational Safety and Health warns that, although AI has the potential to improve risk management, these systems may reproduce and amplify human biases. Such risks can lead to unintended consequences for workers’ health and safety, reinforcing the need for constant oversight and ethical use of these technologies [^7^].

### CHALLENGES AND FUTURE PERSPECTIVES

As technologies continue to evolve, the challenges of preserving human care in occupational health become increasingly evident. The automation of care processes may contribute to the depersonalization of services, creating distance between workers and health professionals. In this context, it is essential that professionals maintain care practices grounded in empathy, respect, and the appreciation of the human dimension, regardless of the level of digital innovation adopted.

The future of occupational medicine therefore depends on achieving a balance between technology and humanity. AI can, and should, be used as a tool to enhance care, increase efficiency, and support decision-making. However, its application must necessarily be complemented by genuine care, sustained by qualified listening and an understanding of the worker as a unique individual.

## CONCLUSIONS

The incorporation of AI into occupational health represents a significant advance, with the potential to improve diagnostic accuracy, optimize resources, and strengthen preventive actions. However, its effective implementation requires recognition that human care is an essential and non-negotiable element. The future of occupational medicine will depend on the ability to integrate technological innovation with practices guided by listening, empathy, and an understanding of the multiple dimensions that shape the working individual.

The qualified presence of health professionals, supported by relational competencies and ethical sensitivity, remains central to the construction of healthier, more equitable, and more sustainable organizational environments. Promoting genuine care, grounded in human values and social responsibility, not only strengthens adherence to health programs but also fosters bonds of trust, belonging, and well-being in the workplace.

Care thus emerges as the link between the future and the present, between innovation and humanity. By adopting organizational models that recognize care as a structural axis, institutions contribute not only to the health of their teams but also to the development of a more empathic, resilient, and humanized organizational culture. This irreplaceable care sustains the construction of a fairer, more balanced, and more sustainable world of work.
